# Intimate partner violence as a predictor of antenatal care service utilization in Honduras

**DOI:** 10.26633/RPSP.2017.104

**Published:** 2017-07-20

**Authors:** Anne K. Sebert Kuhlmann, Janine Foggia, Qiang Fu, Manuel Sierra

**Affiliations:** 1 College for Public Health and Social Justice Saint Louis University, St. Louis Missouri United States of America College for Public Health and Social Justice, Saint Louis University, St. Louis, Missouri, United States of America.; 2 Departamento de Salud Pública Facultad de Ciencias Médicas, Universidad Nacional Autónoma de Honduras Francisco Morazán Honduras Departamento de Salud Pública, Facultad de Ciencias Médicas, Universidad Nacional Autónoma de Honduras, Tegucigalpa, Francisco Morazán, Honduras.

**Keywords:** Intimate partner violence, prenatal care, reproductive health, Honduras., Violencia de pareja, atención prenatal, salud reproductiva, Honduras., Violência por parceiro íntimo, cuidado pré-natal, saúde reprodutiva, Honduras

## Abstract

**Objective:**

*To describe the relationship between exposure to physical and/or sexual intimate partner violence (IPV) and indicators of antenatal care (ANC) service utilization among Honduran women of reproductive age*.

**Methods:**

*Data from the 2011-2012 Honduras Demographic and Health Survey were analyzed to describe the relationship between self-reported exposure to IPV and two ANC outcomes:* (1) *sufficient ANC visits (defined by the Honduran Ministry of Health as five or more visits) and* (2) *early ANC initiation (within the first trimester). Multiple logistic regression was used to estimate effects of physical and sexual IPV on the outcomes, controlling for women’s age, education, literacy, residence, household size, religion, parity, wealth, husband’s age, and husband’s education*.

**Results:**

*Of women who were married, had at least one living child 5 years or younger, and completed the IPV module (N = 6 629), 13.5% of them reported any physical IPV, and 4.1% reported both physical and sexual IPV. There was no significant association between IPV and early ANC; however, a significant relationship between IPV and sufficient ANC was found. Women who experienced any physical IPV (adjusted odds ratios (aOR) = 1.25; 95% confidence interval (CI): 1.00-1.56) or sexual IPV (aOR = 1.53; 95% CI: 1.08-2.16) were, respectively, 25% and 53% more likely to receive insufficient ANC*.

**Conclusions:**

*Honduras has one of highest rates of interpersonal violence of any nation in the world. In Honduras, IPV is a contributor to this broader category of interpersonal violence as well as a risk factor for insufficient ANC. Our findings suggest that universal IPV screening during ANC as well as future initiatives aimed at reducing IPV might improve ANC utilization in the country*.

## RESUMEN

**Objetivo.:**

El presente estudio tuvo por objeto describir la relación entre la exposición a la violencia física o sexual infligida por la pareja y los indicadores de utilización de los servicios de control prenatal por las mujeres hondureñas en edad fecunda.

**Métodos.:**

Se analizaron los datos de la Encuesta de Demografía y Salud del 2011–2012 de Honduras con el fin de describir la relación entre la exposición autonotificada a la violencia de pareja y dos resultados de la atención prenatal, a saber: 1) un número suficiente de consultas de control prenatal (definido como cinco o más por el Ministerio de Salud hondureño) y 2) el inicio temprano del control prenatal (durante el primer trimestre del embarazo). Se aplicó un modelo de regresión logística multivariante a fin de calcular los efectos de la violencia de pareja tanto física como sexual en los indicadores, tras ajustar con respecto a la edad de la mujer, la escolaridad, el alfabetismo, el lugar de residencia, el tamaño del hogar, la religión, el número de partos, el nivel de riqueza, la edad de la pareja y su escolaridad.

**Resultados.:**

De las mujeres casadas, que tenían por lo menos un hijo vivo de 5 años o menor y que completaron el módulo de violencia de pareja de la encuesta (n = 6 629), 13,5% refirieron algún tipo de violencia física y 4,1% notificaron violencia física y sexual infligida por la pareja. No se observó una relación estadísticamente significativa entre la violencia de pareja y el control prenatal temprano; sin embargo, se encontró una asociación significativa entre la violencia de pareja y el número suficiente de consultas de control prenatal. La probabilidad de recibir una atención prenatal insuficiente fue mayor en las mujeres que sufrieron algún tipo de violencia de pareja y, en el caso de la violencia física, fue de 25% (razón de posibilidades ajustadas [ORa]) = 1,25; intervalo de confianza de 95% [IC]: 1,00-1,56) y de la violencia sexual fue 53% (ORa = 1,53; IC de 95%: 1,08-2,16).

**Conclusiones.:**

Honduras tiene una de las tasas más altas de violencia interpersonal de todos los países del mundo. En este país, la violencia de pareja es un factor que contribuye a la categoría más amplia de la violencia interpersonal y representa además un factor de riesgo de tener un control prenatal insuficiente. Los resultados del presente estudio indican que la detección sistemática universal de la violencia de pareja en el marco de la atención prenatal y las iniciativas futuras encaminadas a reducir este tipo de violencia podrían mejorar la utilización del control prenatal en el país.

***Objetivo*.:**

Descrever a relação entre a exposição à violência doméstica física e/ou sexual praticada pelo parceiro íntimo e os indicadores de utilização de serviços de atenção pré-natal entre mulheres hondurenhas em idade reprodutiva.

***Métodos*.:**

Foram analisados dados da Pesquisa de Demografia e Saúde 2011–2012 de Honduras para descrever a relação entre a exposição à violência doméstica praticada pelo parceiro íntimo e dois desfechos da atenção pré-natal: (1) consultas de atenção pré-natal em número adequado (definido pelo Ministério da Saúde hondurenho como cinco ou mais consultas) e (2) início precoce da atenção pré-natal (no primeiro trimestre). Foi usada regressão logística múltipla para estimar os efeitos da violência doméstica física e sexual nos desfechos após controlar para idade, nível de escolaridade, alfabetismo, local de domicílio, tamanho da família, paridade e renda da mulher e idade e nível de escolaridade do parceiro.

**Resultados.:**

Dentre as mulheres casadas, com pelo menos um filho vivo com até 5 anos de idade e que responderam o módulo de violência doméstica (N = 6.629), 13,5% informaram violência física e 4,1% informaram violência física e sexual. Não houve associação significativa entre a violência doméstica e o início precoce da atenção prénatal, porém se verificou uma relação significativa entre a violência doméstica e um número adequado de consultas no pré-natal. As mulheres que vivenciaram violência doméstica física (odds ratio ajustado [aOR] 1,25; intervalo de confiança de 95% [IC 95%] 1.00–1.56) ou sexual (aOR 1,53; IC 95% 1,08–2,16) apresentaram uma chance 25% e 53% maior, respectivamente, de ter atenção pré-natal inadequada.

**Conclusões.:**

Honduras tem uma de taxas mais elevadas de violência interpessoal de todo o mundo. A violência doméstica praticada pelo parceiro íntimo no país é um fator contribuinte à categoria mais ampla de violência interpessoal e constitui um fator de risco para atenção pré-natal inadequada. Os nossos resultados indicam que o rastreamento universal da violência doméstica na atenção pré-natal, aliado a iniciativas futuras para reduzir este tipo de violência, poderia melhorar a utilização de serviços de atenção pré-natal no país.

Intimate partner violence (IPV) is defined by the World Health Organization (WHO) as “any behaviour within an intimate relationship that causes physical, psychological or sexual harm to those in the relationship” (1). IPV is a major global health issue. It is estimated that one in three ever-partnered women experiences physical and/or sexual violence during her lifetime (2). IPV contributes to significant health burden, as women who experience IPV have both more physical and psychological health issues, incur higher health care costs, visit health providers more frequently, and have more hospital stays (2). Specific to women’s health, IPV is a risk factor for poor sexual, reproductive, and maternal health outcomes, such as HIV infection, low-birthweight babies (3), miscarriages (4), and unwanted pregnancies (2, 5–8).

Poor pregnancy outcomes can be minimized through the provision of antenatal care (ANC). Optimal ANC includes initiating care within the first three months of pregnancy, receiving at least four ANC visits, and receiving care from a skilled provider. Because both IPV and ANC are associated with pregnancy outcomes, it is important to understand the relationship between them.

*Intimate partner* violence (IPV) is one type of, and a contributor to, the broader category of *interpersonal* violence. Interpersonal violence, in turn, is one of three broad categories of violence, along with self-directed violence and collective violence. Interpersonal violence refers to intentional harm inflicted by one individual, or a small group of individuals, on another person, usually by people who know each other. This can include family members, intimate partners, friends, and acquaintances (1).

Honduras currently has one of the highest rates of interpersonal violence in the world (9). The country reports a 27% prevalence of lifetime physical interpersonal violence and 10% interpersonal prevalence within the preceding 12 months. Additionally, 20.6% and 3.2% of women report experiencing psychological and sexual violence, respectively, during the preceding 12 months (10).

ANC utilization indicators are relatively high in Honduras, with 89% of women receiving at least four ANC visits, 78% of women initiating care during the first trimester of pregnancy, and 83% of births attended by a skilled provider (10). However, IPV may negatively affect ANC service utilization and influence the relationship between ANC utilization and pregnancy outcomes. Interpersonal violence is the third leading cause of adult mortality in the country (11), so it is critical to understand its interplay with health indicators, specifically ANC service utilization.

To date, nearly all studies describing the relationship between IPV and ANC have been conducted in regions other than Latin America and in countries that are geographically and culturally dissimilar to Honduras. That previous research has indicated that women who experience IPV initiate ANC later (11, 12), are less likely to receive sufficient ANC (13-15), and are less likely to utilize a skilled provider (14–17), as compared to women who have not experienced IPV. Nevertheless, a study of Egyptian women who experienced abuse found that they were actually more likely to receive four or more ANC visits (16). Similarly, a study of 10 Demographic and Health Survey (DHS) countries reported mixed results, suggesting that there are few clear associations between women’s experiences of IPV and seeking ANC in the first trimester or having an institutional delivery (18). These findings also suggest that the relationship may be country specific, with a combination of factors influencing the relationship.

The WHO’s ecological model for violence provides a framework for considering these factors and for organizing them in various levels that radiate outwards from the individual, to relationship characteristics, to the community, and finally, to societal factors (1). For example, age, education, and employment status are individual-level factors considered in nearly all previous research on IPV and ANC (13, 15-18). The husband’s or partner’s age, education, and employment are often considered as part of the relationship characteristics (15, 16), which can also include women’s decision-making autonomy (15) and children in the household (13, 18). Community characteristics frequently include urban vs. rural residence (15, 16, 18). Societal factors can include religion (13, 15, 17), ethnicity (13), beliefs and attitudes about gender roles (16), and even mass media exposure (18, 19), which can be a proxy indicator for women’s autonomy and empowerment. (Women who consume mass media from more sources on a regular basis tend to be more empowered and have more autonomy in their decision-making).

Understanding the relationship between IPV and ANC service utilization, including the influence of various factors within the ecological model, is critical to mitigating the impacts of violence and to optimizing ANC utilization in Honduras. Only limited research has examined this relationship within the context of Latin America (12, 18) or in a country with pervasive interpersonal violence. Therefore, our study aims to describe the relationship between exposure to physical and/or sexual IPV and two key ANC service utilization indicators among women in Honduras who are currently married or living together with a partner and who have at least one child age 5 years or younger. The two indicators are: 1) when ANC was initiated during pregnancy and 2) the total number of ANC visits.

## MATERIALS AND METHODS

### Data sources and sample

Data from the 2011–2012 Honduras Demographic and Health Survey were used. The survey was conducted by the country’s National Institute of Statistics from 26 September 2011 to 20 July 2012, using a stratified two-stage cluster design. At the first stage, enumeration areas (EAs) representing roughly equal population sizes were selected from census files. At the second stage, a sample of households was drawn in each EA selected. Within each department, EAs from the 2001 Honduran census were stratified according to urban or rural residence. Within each EA, 23 475 households were randomly selected, of which 91% were surveyed. A total of 24 414 women aged 15 to 49 from these households were eligible to complete the questionnaire, of which 93% responded (10). The domestic violence module was administered to one randomly selected woman per selected household. Of the 8 332 women who completed the domestic violence module, there were 6 629 women who met our inclusion criteria of being currently married or living together with a partner and having at least one child aged 5 years or younger.

### Outcome measures

ANC service utilization was measured using two indicators: early ANC and sufficient ANC. Early ANC was a binary variable defined as attendance of first ANC visit within the first three months of pregnancy, which is in line with WHO guidelines (20). Sufficient ANC was evaluated through a binary variable in which ANC was considered sufficient if a woman attended at least five ANC visits, which is in line with Honduran Ministry of Health guidelines (21).

### Intimate partner violence

IPV was defined as physical or sexual violence inflicted by a husband or partner. Physical violence was assessed through creation of a binary categorical variable measuring whether a woman experienced any of the following done by her husband or partner: (1) pushing, shaking, or throwing something; (2) slapping; (3) arm twisting or hair pulling; (4) punching with a fist or something harmful; (5) kicking or dragging; (6) choking or burning; and/or (7) threatening or assaulting with a knife, gun, or other weapon. Sexual violence was assessed through a binary variable defined as her saying that her husband or partner had done any of the following: (1) physically forcing her to have sex even though she did not want to, (2) physically forcing her into some other unwanted sexual act, and/or (3) forcing her to perform sexual acts.

### Covariates

Several sociodemographic variables that have been linked to IPV (12, 18) were included in the study. Women’s and husband’s age were included as continuous variables. Women’s and husband’s education (22, 23) was defined according to the highest level completed: (1) no education, (2) primary, (3) secondary, or (4) higher. Women’s illiteracy was defined as not able to read part or all of a sentence, and women’s literacy was defined as able to read a whole sentence. Residence was categorized as rural or urban. Religion was categorized as Catholic or Evangelical/Protestant (which are the main religious affiliations in the country) versus no affiliation or other affiliation. Women’s employment status (24, 25) was defined as currently working outside the home or not. Additionally, household size (15), parity (15), and wealth index (26–28) were included as continuous variables.

We included mass media exposure in our analysis as it has been linked to IPV in several countries (19, 29–31). Mass media exposure was categorized as exposed to no, one, two, or three types of media (i.e., newspaper/magazine, radio, or television) on a weekly basis.

### Statistical analysis

The 2011–2012 Honduras Demographic and Health Survey was a multistage complex sampling survey. Therefore, we incorporated stratification, clustering, and weight variables in all our analyses, which used the survey procedures in SAS version 9.4 software (SAS Institute, Cary, North Carolina, United States of America). The SAS DOMAIN statement was used to analyze the subsample. Student’s *t* test was used to examine the difference of continuous demographic variables between physical and nonphysical IPV as well as between sexual and nonsexual IPV in the bivariate analysis. The chi-square test was used to examine different distributions associated with qualitative variables in the bivariate analysis. Multivariate analysis was performed to examine whether physical or sexual IPV were associated with a greater likelihood of delayed ANC or insufficient ANC, after controlling for other covariates using logistic regression. Odds ratios (ORs) and 95% confidence intervals (CIs) were estimated.

### Ethical considerations

The analyses that we ran used de-identified, secondary data obtained through the DHS request process; those DHS data are publicly and freely available to researchers. This study was reviewed and approved by the human subjects office at Saint Louis University.

## RESULTS

Our sample, on average, was 31.3 years old, with an average parity of 3.2 (Table 1). Most of the women had only a primary-level education (62.3%) but were functionally literate (89.8%). Just under half (43.5%) lived in urban areas, while most (64.4%) were not employed outside the home. A vast majority of the women (94.4%) were exposed to one or more modes of mass media on a weekly basis. Their partners were, on average, slightly older (35.3 years), with about two-thirds (66.0%) having a primary-level education.

**TABLE 1.** Selected sociodemographic characteristics of women who were married and had at least one living child 5 years or younger, from 2011–2012 Honduras Demographic and Health Survey (*N* = 6 629),^a^ used in study of intimate partner violence (IPV) and antenatal care service utilization in Honduras

Characteristic		Any physical IPV		Any sexual IPV	
	Yes *n* (%)		No *n* (%)	Yes *n* (%)	No*n* (%)
Women’s education					
No education	5.0%	72 (5.5)	320 (5.0)	22 (6.8)	370 (5.0)
Primary	62.3%	736 (64.6)	3 644 (61.8)	204 (74.7)	4 175 (61.7)
Secondary	28.0%	261 (25.9)	1 341 (28.4)	42 (15.7)	1 560 (28.5)
Higher	4.7%	34 (4.0)	221 (4.8)	6 (2.8)	249 (4.8)
*P* value^b^			0.27		< 0.001
Husband’s education^c^					
No education	6.3%	73 (6.1)	413 (6.4)	28 (9.9)	458 (6.2)
Primary	66.0%	764 (68.2)	3 802 (65.4)	207 (75.5)	4 358 (65.5)
Secondary	23.1%	234 (21.6)	1 088 (23.4)	37 (13.5)	1 285 (23.5)
Higher	4.7%	32 (4.1)	218 (4.8)	2 (1.1)	248 (4.8)
*P value*			0.52		< 0.001
Women’s literacy^d^					
Fully literate	89.8%	949 (88.0)	4 867 (90.1)	228 (85.9)	5 587 (89.9)
Illiterate	10.2%	150 (12.0)	648 (9.9)	45 (14.1)	753 (10.1)
*P* value			0.07		0.06
Residence					
Rural	56.5%	687 (50.5)	3 830 (57.8)	181 (56.6)	4 335 (56.5)
Urban	43.5%	416 (49.5)	1 696 (42.2)	93 (43.4)	2 019 (43.5)
*P* value			< 0.001		0.98
Religious affiliation^c^					
Catholic/Protestant	89.0%	965 (88.7)	4 932 (89.1)	243 (90.3)	5 653 (89.0)
No or other	11.0%	134 (11.3)	561 (10.9)	29 (9.7)	666 (11.0)
*P* value			0.70		0.48
Women’s employment status^c^					
Employed outside the home	37.0%	660 (59.3)	3 731 (65.4)	153 (55.2)	4 237 (64.8)
Not employed outside the home	63.0%	442 (40.7)	1 789 (34.6)	121 (44.7)	2 110 (35.2)
*P* value			0.001		0.01
Women’s mass media exposure					
No exposure	5.6%	90 (6.5)	363 (5.4)	29 (9.3)	424 (5.4)
Exposed to 1 type	31.9%	396 (31.0)	2 058 (32.1)	109 (35.8)	2 345 (31.8)
Exposed to 2 types	40.1%	430 (40.1)	2 118 (40.1)	102 (38.0)	2 446 (40.2)
Exposed to 3 types	22.4%	187 (22.4)	987 (22.4)	34 (16.9)	1 139 (22.6)
*P* value			0.68		0.05
Women’s age, (yr) (mean)	31.3	29.1	28.9	30.6	28.8
*P* value			0.36		0.002
Husband’s age (yr)^c^ (mean)	35.3	33.4	33.5	36.1	33.4
*P* value			0.78		0.001
Wealth index category (mean)	3.1	2.8	2.8	2.5	2.8
*P* value			0.96		< 0.001
Parity (mean)	3.2	3.1	2.8	3.6	2.8
*P* value			< 0.001		< 0.001
Household size (mean)	5.8	5.4	5.3	5.6	5.3
*P* value			0.08		0.01

a Sample sizes are weighted.

b *P* values for chi-square tests (categorical variables) and Student’s *t* tests (continuous variables).

c Sample size slightly less than the 6 629 total due to missing data.

***Source:*** Table prepared by the authors based on their analyses.

Among our sample, any use of ANC during the most recent pregnancy was nearly 100%. Early initiation of ANC was reported by 5 200 (81.9%) of the women, while 5 443 (83.6%) reported receiving at least five ANC visits during their most recent pregnancy, which was the Government of Honduras’s standard for sufficient ANC at the time of the study. Regarding exposure to IPV, 13.5% of the women reported physical IPV, and 4.1% reported both physical and sexual IPV. No women reported sexual IPV without physical IPV.

In a model adjusting for all covariates, we found no relationship between either reported physical IPV or sexual IPV and delayed initiation of ANC (Table 2). We did, however, find a significant relationship between both exposure to physical IPV and exposure to sexual IPV and receiving an insufficient number of ANC visits. Women who had experienced physical IPV during the preceding 12 months were 25% more likely to get fewer than the recommended five ANC visits, while women who had experienced sexual IPV were 53% more likely to have had insufficient ANC during their most recent pregnancy.

**TABLE 2.** Adjusted odds ratios (aORs)^a^ and 95% confidence intervals (CIs)^b^ for associations between intimate partner violence (IPV) and antenatal care (ANC) utilization among women in Honduras who were married and had at least one living child 5 years or younger, using data from the 2011–2012 Honduras Demographic and Health Survey

IPV	Delayed ANC initiation^c^	Insufficient ANC^d^
	aOR (95% CI)	aOR (95% CI)
Any physical IPV		
Yes	0.91 (0.72–1.16)	1.25 (1.00–1.56)^e^
No	1.00	1.00
Any sexual IPV		
Yes	0.94 (0.64–1.38)	1.53 (1.08–2.16)^e^
No	1.00	1.00

a Models were adjusted for women’s age, husband’s age, women’s education, husband’s education, women’s literacy, area of residence, religion, women’s employment outside the home, wealth, household size, parity, and women’s mass media exposure.

b 95% CIs are from the logistic regression models.

c Delayed ANC initiation = attendance of first ANC visit occurring at 4 months or later.

d Insufficient ANC = receiving fewer than five ANC visits, which is the minimum recommended by the Honduran Ministry of Health.

e *P* < 0.05.

***Source:*** Table prepared by the authors based on their analyses.

Several sociodemographic and household characteristics were also significantly associated with insufficient ANC in both the model for physical IPV and the model for sexual IPV (Table 3) (the physical IPV model is to the left and the sexual IPV model is to the right in the table). In both models, insufficient ANC had a positive association with household size, parity, no or “other” religious affiliation, and the husband having no formal education. However, also in both models, there was an inverse association with women’s age, husband’s age, wealth, and women’s exposure to mass media.

**TABLE 3.** Adjusted odds ratios (aORs)^a^ and 95% confidence intervals (CIs)^b^ for associations that physical intimate partner violence (IPV) and sexual IPV have with insufficient^c^ antenatal care (ANC) utilization among women who were married and had at least one living child 5 years or younger, using data from the 2011–2012 Honduras Demographic and Health Survey

Characteristic	Insufficient ANC	Insufficient ANC
	aOR (95%CI)	aOR (95%CI)
Physical IPV	1.25 (1.00–1.56)^d^	NA^e^
Sexual IPV	NA	1.53 (1.08–2.16)^d^
Women’s education		
No education	2.03 (0.82–5.00)	2.01 (0.82–4.94)
Primary	1.69 (0.77–3.69)	1.68 (0.77–3.67)
Secondary	1.27 (0.58–2.77)	1.27 (0.58–2.76)
Higher	1.00	1.00
Husband’s education		
No education	2.83 (1.29–6.24)^d^	2.80 (1.27–6.17)^d^
Primary	1.84 (0.90–3.60)	1.83 (0.89–3.76)
Secondary	1.78 (0.88–3.60)	1.78 (0.88–3.59)
Higher	1.00	1.00
Women’s illiteracy	1.09 (0.80–1.49)	1.10 (0.81–1.50)
No or other religious affiliation	1.52 (1.18–1.95)^d^	1.52 (1.18–1.96)^d^
Household size	1.05 (1.00–1.10)^d^	1.05 (1.00–1.10)^d^
Parity	1.31 (1.22–1.41)^d^	1.32 (1.23–1.41)^d^
Women’s age, years	0.95 (0.93–0.97)^d^	0.95 (0.93–0.97)^d^
Husband’s age, years	0.99 (0.98–1.00)^d^	0.99 (0.98–1.00)^d^
Rural residence	0.85 (0.66–1.10)	0.85 (0.66–1.09)
Women not employed outside the home	0.92 (0.76–1.11)	0.92 (0.76–1.12)
Wealth index	0.85 (0.76–0.95)^d^	0.85 (0.76–0.95)^d^
Women’s mass media exposure	0.86 (0.76–0.97)^d^	0.86 (0.77–0.97)^d^

a Models were adjusted for women’s age, husband’s age, women’s education, husband’s education, women’s literacy, area of residence, religion, women’s employment status, wealth, household size, parity, and women’s mass media exposure.

b 95% CIs are from the logistic regression models.

c Insufficient antenatal care = receiving fewer than five ANC visits, the minimum recommended by the Honduran Ministry of Health.

d *P* < 0.05.

e NA = not applicable.

***Source:*** Table prepared by the authors based on their analyses.

## DISCUSSION

To the best of our knowledge, ours is the first study analyzing the relationship between IPV and ANC service utilization in Honduras, and one of the few such studies published from anywhere in Latin America. The issue of IPV and reproductive health service utilization and outcomes is particularly important to explore within the context of pervasive interpersonal violence, which is currently plaguing several countries of Central America and the rest of Latin America. The DHS provides one of the few sources of publicly available, population-based data with reproductive health indicators; exposure to IPV is the only form of interpersonal violence measured in the DHS household questionnaire.

Overall, self-reported ANC service utilization rates in Honduras are higher than in many other low- or lower-middle-income countries. Higher ANC rates may reflect higher overall use of health services in Honduras, as exemplified by the Ministry of Health having a standard of care for sufficient ANC set at five visits (21), which is higher than the WHO’s recommended standard of four visits (20). The number of visits attended does not, however, provide any indication of quality of care received during those visits, nor does it reflect health outcomes. Despite high ANC service utilization, Honduras has a maternal mortality ratio nearly twice that of the Latin American and Caribbean region overall, and a relatively high perinatal mortality rate (11). As the Ministry of Health has decentralized responsibility for health service provision to nongovernmental organizations throughout the country, quality and consistency of care within the system are issues that require further investigation.

While ANC utilization appears to be higher in Honduras than in some other low- or lower-middle-income countries, reported exposures to physical IPV (13.5%) and to sexual IPV (4.1%) in Honduras are lower than in similar studies from elsewhere. Physical IPV has been reported by 34% of married Egyptian women (16), approximately 48% of Bangladeshi women (15), and, during pregnancy, by nearly 23% of rural Indian women (13). A nine-country study of DHS data from the late 1990s and early 2000s reported ever-exposure to physical IPV ranging from a low of 17.5% in Cambodia to a high of 48.4% in Zambia. The two Latin American countries included in the analysis, Nicaragua (30.2%) and the Dominican Republic (22.3%), each reported substantially higher levels than women in Honduras during 2011–2012 (12). Similarly, exposure to sexual IPV ranges from a low of 3.6% in Cambodia to a high of 17.0% in Haiti, with the Dominican Republic and Nicaragua reporting 6.4% and 10.2%, respectively (12). A later DHS analysis of 10 countries (some overlapping) found reports of physical IPV to range from 12% in Haiti to 71% in Bangladesh and sexual IPV ranging from 3% in Moldova to 26% in Bangladesh (18). The lower IPV rates reported by women in Honduras may reflect that IPV truly is lower there than in many other developing countries, including others in Latin America.

Alternatively, within the context of pervasive societal interpersonal violence in Honduras, these lower rates of IPV may reflect a fear of reporting IPV and/or a tolerance or acceptance of less dramatic levels of violence (e.g., slapping, punching) as normal. General societal acceptance of physical violence against women by their husbands or partners has been documented (32). One study from Serbia found that women often did not report abuse by their husband or partner because they considered it bearable (33). More research is needed to understand IPV within the context of pervasive societal interpersonal violence in Honduras.

In bivariate analyses, several sociodemographic and household characters—particularly ones that point to women’s status and empowerment—were significantly associated with physical and/or sexual IPV. Our findings showing that women exposed to more forms of mass media on a weekly basis reported less IPV are consistent with previous findings from elsewhere (19). Interestingly, we found an inverse relationship between wealth and sexual IPV but not physical IPV. That contrasts with several other studies that have found such a relationship (12, 26–28). Not surprisingly, husbands’ characteristics, such as age and education level, were associated with sexual IPV, similar to findings from elsewhere (12, 13, 22, 23). Future studies should consider additional sociodemographic characteristics such as use of alcohol, family history of violence, and depression in women.

A recent randomized trial from northern Ecuador reported that interventions to transfer cash, vouchers, or food to women—irrespective of the modality of the transfer—decreased rates of physical and sexual IPV. This may have been due to changing the power dynamic in the household and reducing women’s economic reliance on their husbands (34). Several similar conditional cash-transfer interventions are currently being implemented in Honduras (35). These interventions could help increase women’s status and empowerment and decrease IPV, while simultaneously addressing other health issues linked to poverty, such as malnutrition. However, whether IPV is being measured as a component of the evaluations of these interventions in Honduras remains unclear.

Despite several studies that have found IPV to be an important determinant of delayed initiation of ANC (13, 18), our model that was adjusted for all covariates failed to find a significant relationship between either physical IPV or sexual IPV and delayed ANC. Again, this may reflect the existence of societal norms for service utilization in general in Honduras that are strong enough such that exposure to IPV is not a major barrier to women initiating ANC in a timely fashion. Early ANC does not, however, necessarily equate to better ANC, nor does it indicate utilization of other sexual and reproductive health services such as HIV testing or contraceptive use. There is still much to understand in the relationship between IPV and utilization of sexual and reproductive health services, especially in lower-resource settings in which people are exposed to pervasive societal interpersonal violence.

We did find that both physical IPV and sexual IPV had a relationship with insufficient ANC. These results are consistent with other studies, from both developed countries (36) and developing nations (15), that have found IPV to be an important determinant of reproductive health service utilization. Specifically, multiple studies have found a relationship with insufficient ANC (13, 15), consistent with our findings. At least one study, however, found a significant relationship in the opposite direction: Egyptian women who experience IPV utilize *more* ANC (16). Given that Egypt is geographically and culturally different from Honduras, there may be important factors along the various levels of the ecological model that influence the relationship between IPV and ANC service utilization. As with studies from elsewhere, covariates representing several levels of the ecological model were also significant in our study. These included women’s age and parity at the individual level; husband’s age and education, household size, and wealth index at the relationship level; and exposure to mass media at the societal level. Interestingly, women’s education, literacy, and employment status were not significant in either model, nor was rural residence.

Based on these findings, we recommend more research on IPV and reproductive health service utilization and outcomes in low-resource settings, especially within the context of pervasive societal interpersonal violence. Incorporating universal screening for IPV as part of a woman’s first ANC visit could be an important step towards mitigating the consequences of IPV. There is also some evidence from high-income settings that the screening assessment itself can serve as an intervention because it signals to women that the issue is serious, that the health care provider is concerned about the issue, and that resources might be available to help (37). Also, evaluating the impact of conditional cash transfer programs on IPV could identify these programs as primary prevention interventions that both reduce IPV and improve ANC. Finally, we need to explore whether and how IPV may influence utilization of other reproductive health services that have a lower overall utilization rate in the population, such as HIV testing and contraceptive services.

### Strengths and limitations

The DHS methodology is well known, tested, and documented. This enhances confidence in the data, especially when looking at a potentially sensitive issue such as IPV. The nationally representative sample from the 2011-2012 Honduras DHS that completed the domestic violence module is relatively large (over 6 660) and is larger than in similar analyses from countries elsewhere that have a larger populations (13, 15, 18).

Despite these strengths, the results must be interpreted with caution. The analyses rely on cross-sectional survey data, and so the temporality between the exposure to IPV and the most recent pregnancy cannot be established. Furthermore, given the nature of secondary analysis, we were limited to indicators of IPV and thus could not look at the broader issue of exposure to interpersonal violence. Similarly, the items in the domestic violence modules of the DHS are not tailored to the country context, so that the questions about IPV are not situated within this broader context of violence. Finally, the relatively low reporting of IPV in our study, especially in comparison to other Latin American countries, suggests the potential for social desirability bias. In addition, there might be reluctance to report IPV within the context of pervasive societal violence, such as Honduras is currently experiencing, despite the standard DHS methodology of training surveyors to collect this data sensitively.

### Conclusions

We described the relationship between physical and sexual IPV and the ANC service utilization indicators of early ANC initiation and sufficient ANC visits. We found no significant relationship between either physical or sexual IPV and early ANC initiation, but we did find significant relationships between both physical and sexual IPV and insufficient ANC visits, after controlling for a number of sociodemographic characteristics. Consistent with previous studies from elsewhere, several individual and relationship characteristics were significant in both models for insufficient ANC visits in Honduras.

### Disclaimer.

Authors hold sole responsibility for the views expressed in the manuscript, which may not necessarily reflect the opinion or policy of the *RPSP/PAJPH* or PAHO.
